# The Contemporary Role of Intracoronary Physiological Assessment: Fractional Flow Reserve, Non-Hyperemic Pressure Ratios, Wireless Technologies, and Microcirculation

**DOI:** 10.3390/jcdd13070300

**Published:** 2026-07-01

**Authors:** Andreas S. Triantafyllis, Sotirios C. Kotoulas, Iosif Xenogiannis, Leonidas E. Poulimenos, Ignatios Ikonomidis, Andreas S. Kalogeropoulos

**Affiliations:** 1 Department of Cardiology, “Asklepeion” General Hospital of Voula, 16673 Athens, Greece; soter96@med.uoa.gr (S.C.K.); kardio@asklepieio.gr (L.E.P.); 2 Department of Cardiology, Attikon University Hospital, 12462 Athens, Greece; ixenogiannis@med.uoa.gr (I.X.); igikon@med.uoa.gr (I.I.); 3 Department of Cardiology, Hygeia Hospital HHG, 15123 Athens, Greece; akalogeropoulos@hygeia.gr

**Keywords:** fractional flow reserve, instantaneous wave-free ratio, non-hyperemic pressure ratios, quantitative flow ratio, coronary microvascular dysfunction, INOCA, coronary physiology, interventional cardiology

## Abstract

**Background/Objectives**: Angiographic stenosis severity and functional significance are discordant in up to 65% of intermediate coronary lesions. Fractional flow reserve (FFR)-guided percutaneous coronary intervention (PCI) has shown better clinical outcomes than standard angiography-guided PCI, therefore functional significance defines revascularization. This review evaluates the contemporary evidence for intracoronary physiology assessment tools, such as FFR, non-hyperemic pressure ratios (NHPRs), angiography-derived wire-free indices, and microvascular function testing, and proposes a framework for their implementation into clinical practice. **Methods**: We conducted a narrative review, synthesizing data from landmark randomized controlled trials (DEFER, FAME I–III, DANAMI-3-PRIMULTI, COMPARE-ACUTE, DEFINE-FLAIR, iFR-SWEDEHEART, iMODERN, FAVOR III China and Europe, FAST III, ALL-RISE, CorMicA), along with pooled analyses, meta-analyses, position papers, and relevant guidelines. **Results**: FFR-guided revascularization resulted in a 28% reduction in cardiac death or myocardial infarction in pooled analyses (HR 0.72, 95% CI 0.54–0.96). leading to a Class I, Level A indication. NHPRs, including iFR, achieved non-inferiority to the FFR at 1 year; however, a 5-year pooled meta-analysis raised concerns of increased all-cause mortality with iFR guidance compared to the FFR (HR 1.34, 95% CI 1.08–1.67). Approximately 20% of lesions show FFR–iFR discordance, driven by vessel-specific physiology and microvascular factors. Wire-free technologies yielded conflicting results: the FAVOR III China trial favored the QFR over angiography, yet FAVOR III Europe failed non-inferiority versus the FFR, while the recent FAST III and ALL-RISE trials demonstrated the non-inferiority of angiography-derived physiology at 1 year. Up to 40% of patients with angina have non-obstructed coronary arteries, and coronary vasomotor function testing can identify treatable microvascular endotypes improving symptoms and quality of life. **Conclusions**: Functional invasive coronary angiography is advocated to decipher vessel hemodynamics and to guide treatment. The FFR remains the gold standard for invasive physiological assessments, while NHPRs and wire-free technologies are valuable adjuncts with specific indications and limitations. A thorough microvascular evaluation is essential for differentiating between various INOCA endotypes and is gradually being adopted by the interventional community. While NHPRs and virtual technologies struggle to dethrone the king FFR, a comprehensive intracoronary physiology assessment is essential to guide treatment.

## 1. Introduction

Coronary angiography (CA) has been the cornerstone for the diagnosis of coronary artery disease (CAD) since its introduction by Mason Sones in 1958 [1]. Its main strength is the direct visualization of the contrast-filled coronary lumen by providing a two-dimensional luminogram. However, CA cannot reliably define vessel wall pathology, plaque burden, or the physiological significance of a stenosis, which attenuates its ability to decipher the complex interplay between plaque, lumen size, and ischemia in CAD [2].

In particular, Glagov’s phenomenon, defined as compensatory vascular positive remodeling, is characterized by outward expansion of the coronary arteries during early stage atherosclerosis, maintaining luminal patency despite the presence of plaque burden [3]. As a result, an angiographically normal vessel may conceal substantial atherosclerotic disease. A visual estimation of stenosis severity is also affected by eccentric lesion morphology, foreshortening, vessel overlap, and diffuse disease without a normal reference segment [4]. These factors reinforce the notion that an anatomical assessment alone is insufficient for clinical decision-making.

The mechanisms of myocardial ischemia in chronic coronary syndromes are multifactorial and extend well beyond focal epicardial obstruction. As per the 2024 European Society of Cardiology (ESC) Guidelines on Chronic Coronary Syndromes, the main mechanisms include both epicardial and microvascular mechanisms [5]. Epicardially, ischemia may result from structural abnormalities, including focal or diffuse atherosclerosis, stabilized intramural hematoma, myocardial bridging, or coronary aneurysm, as well as functional disorders, such as vasospasm related to endothelial dysfunction or vascular smooth muscle hyperactivity. At the microvascular level, ischemia may reflect structural disease, including inward arteriolar remodeling, capillary rarefaction, perivascular fibrosis, and extramural compression, or functional abnormalities, such as impaired vasodilation, endothelial dysfunction, and impaired vasoconstriction [5,6]. Different pathophysiological mechanisms may frequently coexist. Accurate identification is clinically important, because treatment differs substantially across epicardial obstruction, vasospasm, and microvascular dysfunction. CA alone cannot distinguish these mechanisms, which is why a physiological assessment is often necessary for a complete lesion evaluation [7].

The visual assessment of angiographic severity and physiological significance are discordant in approximately 65% of patients with intermediate stenoses [8]. In lesions with 50% to 70% diameter stenosis, the fractional flow reserve (FFR) values range widely from 0.3 to 1.0, and only about 35% are functionally significant using the established threshold of ≤0.80. Even among lesions visually graded as 71% to 90% stenoses, approximately 20% have an FFR >0.80. This discordance has direct clinical consequences: angiography may over- or underestimate lesion significance and subsequently falsely affect the decision to perform or defer revascularization.

A lesion-specific physiological assessment has consistently improved clinical outcomes over angiography-guided decision-making across the DEFER and FAME family of trials, establishing the principle that functional significance rather than anatomical appearance should define revascularization. The FFR remains the gold standard for invasive physiological assessment, supported by Class I, Level A recommendation [5]. However, its dependence on pharmacological complexity, patient discomfort, time, cost, and variable hyperemic responses limit its real-world uptake. These limitations have led to the development of non-hyperemic pressure ratios (NHPRs) and angiography-derived wire-free technologies as practical alternatives. Beyond epicardial disease, 40% of patients with angina and positive non-invasive stress tests are found to have non-obstructed coronary arteries at angiography. These patients carry a meaningful burden of symptoms and present adverse cardiovascular events. Targeted microvascular function testing, as demonstrated in the CorMicA trial, can identify treatable endotypes and improve quality of life [9]. This review synthesizes the contemporary evidence for the full spectrum of intracoronary physiological assessment tools and proposes a framework for their integration into clinical practice, while it is differentiated from contemporary guidelines and statement documents in three respects. Firstly, it incorporates pivotal evidence that postdates or runs concurrent with the aforementioned documents. Secondly, it integrates epicardial, angiography, and computed tomography-derived, as well as microvascular assessment within a single practical decision framework. Lastly, it argues explicitly that angiography-derived indices should not be treated as a single interchangeable class, a distinction not yet reflected in the current guidelines. The aim is to provide an integrated and current synthesis to support decision-making rather than to restate existing recommendations.

## 2. Methods

We performed a narrative review using PubMed/MEDLINE as the primary database. The literature search covered data from 1993 (introduction of the FFR) to May 2026. The search was designed to identify the studies evaluating invasive and non-invasive coronary physiological assessments, including fractional flow reserve, non-hyperemic pressure ratios, angiography-derived indices, computed tomography-derived FFR, and coronary microvascular function testing. The following terms were used alone and in combination: “fractional flow reserve”, “FFR”, “instantaneous wave free ratio”, “iFR”, “non hyperemic pressure ratio”, “resting full cycle ratio”, “diastolic hyperemia free ratio”, “quantitative flow ratio”, “QFR”, “FFRangio”, “vessel FFR”, “CT FFR”, “coronary flow reserve”, “index of microcirculatory resistance”, “coronary microvascular dysfunction”, “vasospastic angina”, “INOCA”, “ANOCA”, and “coronary physiology”. Studies were eligible if they reported original data and randomized controlled trials, large prospective registries, pooled analyses, meta-analyses, or expert consensus statements, and international guideline documents were prioritized. Particular emphasis was given to landmark studies that directly influenced clinical practice, including the DEFER and FAME family of studies, DANAMI 3 PRIMULTI, COMPARE ACUTE, DEFINE FLAIR, iFR SWEDEHEART, iMODERN, FAVOR III China, FAVOR III Europe, FAST III, ALL RISE, CorMicA, and ILIAS ANOCA. Reference lists of the selected articles were also screened to identify additional relevant publications. Given the narrative scope of this review, no formal systematic review protocol, risk of bias assessment, or meta-analytic pooling was undertaken. Throughout this review, the diagnostic performance of NHPRs is expressed relative to the FFR as the established clinical reference standard. The FFR itself is validated against non-invasive markers of ischemia. The comparisons discussed should be interpreted as an agreement between modalities rather than as validation against absolute truth.

## 3. Fractional Flow Reserve

### 3.1. Physiological Principles

The FFR was introduced by Nico Pijls and Bernard De Bruyne in 1993 [10,11]. It represents the ratio of maximal achievable blood flow to a myocardial territory in the presence of a stenosis to the maximum achievable flow in the absence of stenosis. The FFR is calculated as the ratio of two pressures. During maximal hyperemia, coronary flow and coronary pressure (Pd/Pa) achieve a linear correlation, as coronary resistance is kept stable and minimal. By translating the coronary flow into a pressure ratio, the FFR becomes independent of hemodynamic conditions.

The FFR is calculated as FFR = (Pd − Pv)/(Pa − Pv), where Pd is the distal coronary pressure measured by a pressure guidewire placed distal to the stenosis, Pa represents the proximal aortic pressure recorded at the guide catheter tip, and Pv represents the central venous pressure [12]. During maximal hyperemia, it is assumed that Pv reaches zero, so the formula is simplified to FFR ≈ Pd/Pa. This pressure-based simplification is the key innovation that made the FFR clinically practical, by avoiding the need for a direct coronary flow measurement. An FFR value of ≤0.80 is the accepted threshold for hemodynamically significant stenosis, and indicates a high probability of lesion-specific ischemia [13].

Maximal hyperemia can be achieved with adenosine, papaverine, or regadenoson [14]. Adenosine remains the most commonly used agent in routine practice. It can be administered as a continuous intravenous (IV) infusion, usually at 140 μg/kg/min through a central or large peripheral vein, or as an intracoronary bolus, commonly 100–200 μg in the left coronary artery or 50–100 μg in the right coronary artery. Hyperemia reduces coronary autoregulation and brings microvascular resistance to a low and relatively constant level, allowing pressure to approximate flow. IV adenosine is generally preferred when pressure pullback is required, whereas intracoronary administration is a practical option for single lesion assessment, even though it does not allow for a continuous pressure pullback curve [15].

### 3.2. Evidence Base: The FAME Trilogy and Beyond

The clinical evidence for FFR-guided revascularization was built mainly on the landmark DEFER and FAME trial programs, which established the superiority of physiology-guided over angiography-guided vascularization.

The DEFER study, published by Bech et al. in 2001, was the first randomized trial to demonstrate that deferral of percutaneous coronary intervention (PCI) for intermediate stenoses with an FFR > 0.75 was safe [16]. At a 15-year follow-up, event rates remained low in the deferred group, with no excess in death or myocardial infarction compared to revascularized patients [17], providing the earliest evidence that non-ischemic lesions do not benefit from intervention.

The FAME I study, published in 2009, randomized 1005 patients with multivessel coronary artery disease to angiography-guided versus FFR-guided PCI [18]. The FFR-guided approach resulted in a reduced number of implanted stents (1.9 ± 1.3 vs. 2.7 ± 1.2, *p* < 0.001), and improved the clinical outcomes with a significantly reduced composite endpoint of death, myocardial infarction, and repeat revascularization at one year (13.2% vs. 18.3%, *p* = 0.02). This benefit was maintained at two years and was accompanied by lower resource use and improved cost-effectiveness [19].

In the FAME 2 study, 1220 patients were enrolled with at least one stenosis and an FFR ≤ 0.80, randomizing them to PCI OMT versus OMT alone [20]. The study was prematurely terminated due to a significantly lower rate of urgent revascularization in the PCI group. The 5-year follow-up showed a sustained reduction in the composite of death, myocardial infarction, or urgent revascularization with FFR-guided PCI (13.9% vs. 27.0%, *HR* 0.46, 95% *CI* 0.34–0.63, *p* < 0.001) [13,21]. The benefit was driven mainly by a reduction in urgent revascularizations, with a contribution from myocardial infarction reduction, but without a clear mortality advantage.

A pooled analysis by Zimmermann et al., of FAME-2, DANAMI-3-PRIMULTI, and COMPARE-ACUTE, further strengthened the evidence base. In this analysis, PCI of lesions with an FFR ≤ 0.80 reduced cardiac death or myocardial infarction compared to medical therapy *(HR* 0.72, 95% *CI* 0.54–0.96, *p* = 0.023), providing strong evidence for a hard endpoint benefit of FFR-guided revascularization [22].

The FAME 3 study addressed a different question: whether FFR-guided PCI could match coronary artery bypass grafting (CABG) in patients with three-vessel disease [23]. At one year, FFR-guided PCI did not meet non-inferiority compared to CABG (10.6% vs. 6.9%, *HR* 1.5, 95% *CI* 1.1–2.04, *p =* 0.35 *for non-inferiority*). Longer follow-up showed a persistent numerical trend favoring CABG (*HR* 1.16, 95% *CI* 0.89–1.52, *p* = 0.27), although the difference was not statistically significant. At the 5-year follow-up, there was no significant difference in a composite endpoint of death, stroke, or myocardial infarction of FFR-guided PCI vs. CABG, even though the myocardial infarction and repeat revascularization rates were higher with PCI.

### 3.3. FFR as a Continuous Prognostic Marker

Beyond the commonly used threshold of 0.80, the FFR provides continuous prognostic information. Johnson et al., in a pooled analysis of the FAME I and II studies, demonstrated that the relationship between the FFR and clinical outcomes is not binary but gradual. The expected benefit of PCI over deferral, measured as a reduction in major adverse cardiac events (MACE) at two years was greatest at an FFR *≤* 0.60, attenuated progressively as the FFR approached 0.75–0.78, and neutralized at approximately 0.80. Above this threshold, revascularization conferred no benefit and carried unnecessary procedural risk. Lower FFR values therefore indicate not only a higher ischemic burden but a greater absolute likelihood of clinical benefit from intervention [24,25].

The same principle applies post-PCI. Post-procedural FFR carries significant prognostic data, since patients with lower post-PCI FFR values have higher rates of major adverse cardiac events during follow-up. In the DEFINE PCI trial, a post-PCI iFR ≥ 0.95 was associated with significantly lower cardiac death, spontaneous myocardial infarction, or clinically-driven target vessel revascularization at 1 year. Similar findings have been reported for post-PCI FFR with a threshold of ≥0.90 predicting lower MACE at 1–3 years in the DKCRUSH VII registry and across multiple pooled analyses [26,27]. A satisfactory angiographic result does not guarantee an optimal physiological result. Residual diffuse disease, untreated serial lesions, or suboptimal stent expansion may leave a persistent pressure gradient despite an apparently satisfactory final angiogram [28].

The prognostic value of the FFR as a continuous variable was supported by the IRIS FFR registry of 5846 patients. Among deferred lesions, the risk of MACE increased progressively as the FFR decreased (*adjusted HR* 1.06 *per* 0.01 *decrease*, 95% *CI* 1.05–1.08, *p* < 0.001). Revascularization was associated with better outcomes than deferral when the *FFR* ≤ 0.75 (*adjusted HR* 0.47, 95% *CI* 0.26–0.84, *p* = 0.012), whereas medical treatment appeared equally safe for lesions with an FFR ≥ 0.76 [29]. The results indicate that the FFR thresholds are clinically useful but they should not obscure the graded relationship between physiology and risk.

### 3.4. Practical Considerations and Pitfalls

An accurate FFR measurement requires attention to technique at every stage of the procedure. Errors may occur during preparation with calibration and equalization, during measurement with pressure drift, guide catheter wedging, wire whipping and suboptimal hyperemia achievement, or during interpretation with inconsistent cursor positioning [15].

Pressure drift is particularly important because it may not be apparent during measurement. Hence, the pressure wire should be withdrawn to the guide catheter tip after measurement to confirm that the distal and proximal pressures remain equal. Guide catheter-related artifacts are another frequent source of error. Aortic ventricularization is characterized by the loss of the dicrotic notch and deep diastolic dipping results in a lower mean aortic pressure (Pa). Since the FFR is calculated by Pd/Pa, an artificially reduced Pa raises the calculated ratio and produces a falsely high FFR value (false-negative), leading to underestimation of lesion severity [30]. The same issue may occur with deep guide engagement or catheter dampening due to reduction of the aortic pressure signal, thus producing a falsely elevated FFR value. When these artifacts are seen, the guide catheter should be disengaged and the measurement replaced.

Adenosine is generally considered safe, though transient adverse effects are common. The most relevant events and their reported rates are summarized in Table 1, along with pooled estimates from a contemporary meta-analysis [31,32]. Adenosine should be avoided in patients with severe asthma or high degree atrioventricular block without a pacemaker. Its hemodynamic effect varies between patients. In a large prospective cohort, the change in mean aortic pressure during adenosine ranged from a 75% decrease to a 43% increase, with a median decrease of 9% [33]. Inadequate hyperemia may therefore affect the FFR interpretation and has been linked to anatomical and functional mismatch, as well as discordance between low NHPR values and high FFR values [34]. Genetic variation in adenosine metabolism and receptor sensitivity has been proposed as one possible hypothesis, yet this remains mechanistic, and is not a firmly established determinant in routine FFR practice.

### 3.5. Limitations of the FFR

Despite its robust evidence base, the FFR has important practical and physiological limitations. Firstly, achieving maximum hyperemia is a prerequisite. Use of adenosine increases costs, can be time consuming, and bears the risk of side effects (Table 1). The intravenous administration of adenosine mandates for a large vein, while intracoronary administration is a useful alternative but does not allow for pullback. Polymorphisms in genes for endothelial synthase and hemoglobin oxygenase, smoking, chronic kidney disease, or increased microvascular resistance may impair maximal vasodilatation after adenosine administration, leading to incorrect measurements [35].

Its routine interpretation relies on two assumptions: that venous pressure is negligible and that microvascular resistance is minimal and stable during maximal hyperemia [36]. Those assumptions are acceptable in the majority of patients. The standardization document by Toth et al., supports the practical use of the simplified Pd/Pa formula, even in patients with heart failure (HF) and elevated filling pressures, and does not recommend routine correction for right atrial pressure [15].

Nevertheless, an elevated central venous pressure (CVP) does have a directional effect on the simplified formula. When Pv is increased, as in acute HF or cardiogenic shock, the simplified Pd/Pa formula may overestimate the true myocardial FFR (FFRMyo), producing a falsely reassuring result [37,38]. Toth et al. incorporated the right atrial pressures in >1600 coronary stenosis [37]. In 9% of those, the FFR moved from >0.80 to an FFRmyo ≤ 0.80 once the right atrial pressure was incorporated. The absolute difference was no greater than 0.03, and the overall agreement between the FFR and FFRmyo remained excellent, with a mean difference 0.01. Thus, right atrial pressure correction is not needed in routine clinical practice, but markedly elevated filling pressures should be recognized as a potential source of borderline misclassification.

Coronary microvascular dysfunction (CMD) is a clinically relevant limitation. When the vasodilatory capacity is impaired, hyperemic microvascular resistance remains high, coronary flow augmentation is blunted, and the pressure gradient across an epicardial stenosis may be attenuated. The FFR may appear pseudonormal (false-negative) despite clinically important disease. Van de Hoef et al. demonstrated that, for similar epicardial stenosis severity, the FFR increased as the hyperemic microvascular resistance increased, Adjustment for the hyperemic microvascular resistance strengthened the relationship between the FFR and stenosis resistance *(r*^2^ *from* 0.54 *to* 0.73). In the same study, a substantial 63% of vessels with an FFR > 0.80 presented disturbed hemodynamics, including an abnormal CFR in 52%, and microcirculatory dysfunction in 33% [39].

Several patient-related risk factors may influence the hyperemic response. Advanced age is one well-supported example. Age-related increases in the minimal microvascular resistance blunt hyperemic flow augmentation and the FFR may underestimate the hemodynamic effect of a stenosis [40]. Similar concerns apply in patients with left ventricular hypertrophy and severe aortic stenosis, where microvascular remodeling and increased extravascular forces may blunt hyperemia. Diabetes mellitus is more complex. In the POST IT registry of 1772 patients, Van Belle et al. found that diabetes was not associated with higher FFR values for a given angiographic stenosis. In fact, the FFR values were lower in patients with diabetes across the stenosis categories, reflecting more complex epicardial disease [41]. Diabetes alone should not be assumed to invalidate the FFR. Chronic kidney disease and heavy smoking are biologically plausible contributors to microvascular dysfunction but have less direct evidence that they cause clinically meaningful FFR pseudonormalization compared to age, left ventricular hypertrophy, or severe aortic stenosis.

The FFR grey zone between 0.75–0.80 remains a well-recognized clinical dilemma. A systematic review and metanalysis by Andreou et al., including 2683 lesions in this range, found no overall advantage of revascularization over deferral for study-defined MACE, although target vessel revascularization was more frequent with deferral (9.1% vs. 5.8%, *p* = 0.04) [42]. Decisions in the patients with this range of FFR values should depend on lesion location, territory at risk, focal versus diffuse gradient pattern as assessed by pullback, symptom burden, quality of life, viability, bleeding risk, comorbidities, and patient preference [43,44].

Additionally, the ability of the FFR to localize the principal source of hemodynamic significance in patients with diffuse disease or serial lesions is limited. A single FFR measurement distal to multiple stenoses reflects the cumulative physiological effect but does not identify which lesion contributes most to flow limitation, hampering treatment decisions in complex anatomy. A pressure pullback gradient (PPG) can add important information by distinguishing focal from diffuse disease, in patients with an *FFR* ≤ 0.80. Higher values, approaching 1, suggest a focal pressure drop that is more likely to respond well to PCI. Lower values, closer to 0, suggest diffuse disease where intervention may yield suboptimal physiological results and greater risk of residual ischemia or periprocedural injury. Collet et al., described the PPG as a continuous metric and cautioned against rigid cutoffs [45]. Specific operating ranges have been proposed in later studies, describing 0–0.47 as an indicator of diffuse disease and 0.65–1.00 as more consistent with focal/serial disease [46], while Sakai et al. used a median value of 0.66 in their cohort [47]. The PPG should be interpreted as a descriptor of disease pattern and not as a standalone binary treatment threshold.

### 3.6. FFR in Acute Coronary Syndromes

The role of the FFR in acute coronary syndromes is subject to important physiological limitations in the immediate post-infarction period. In STEMI, microvascular obstruction, impaired hyperemic response, and increased microvascular resistance in the culprit territory can attenuate the pressure gradient across non-culprit stenoses, producing falsely reassuring FFR values. This reflects acute microvascular stunning rather than true absence of ischemia, and limits the reliability of the FFR measurements obtained during the index primary PCI procedure. Accordingly, the ESC Guidelines advocate against an FFR-guided assessment of non-culprit lesions in STEMI during the index procedure (Class III) [48], and should be delayed until microvascular function has recovered, typically several days after the acute event. The physiological context differs in NSTEMI, where microvascular function is generally preserved and the FFR can be performed during the index procedure [48,49]. In line with this, the current ESC guidelines recommend that an invasive functional assessment should be considered to guide non-culprit lesion treatment during the index hospitalization in NSTEMI patients with multivessel disease (Class IIb) [5,48].

Several landmark trials have evaluated FFR-guided complete revascularization in STEMI with multivessel disease using a staged approach. In the DANAMI-3-PRIMULTI study, patients who had undergone successful primary PCI were randomized to an FFR-guided complete revascularization of non-culprit lesions or infarct-related artery treatment alone, with an FFR assessment performed after the acute phase [50]. At the 10-year follow-up, the FFR-guided complete revascularization reduced future cardiovascular events compared with infarct-related artery-only treatment (HR 0.76, 95% CI 0.60–0.94, *p* = 0.014), a benefit driven primarily by fewer repeat revascularizations rather than a reduction in death or recurrent myocardial infarction [51]. The COMPARE-ACUTE study similarly employed a staged FFR assessment strategy rather than immediate evaluation during primary PCI [52]. The FLOWER-MI trial added further context, demonstrating that FFR-guided and angiography-guided strategies for non-culprit lesion revascularization produced comparable clinical outcomes in STEMI patients [53]. The most recent and largest trial in this space, the FULL REVASC study, enrolled 1542 patients with STEMI or very-high-risk NSTEMI and multivessel disease, randomizing them to FFR-guided complete revascularization of non-culprit lesions or culprit-lesion-only PCI [54]. At a median follow-up of 4.8 years, the composite of death from any cause, myocardial infarction, or unplanned revascularization did not differ between the groups (19.0% vs. 20.4%; HR 0.93, 95% CI 0.74–1.17; *p* = 0.53), with no between-group differences in either component outcome. The trial evidence suggests that, while an FFR-guided staged assessment of non-culprit lesions is physiologically sound and procedurally feasible in the post-ACS setting, its incremental benefit over selective or angiography-guided approaches remains uncertain and falls short of the consistent advantage demonstrated in stable coronary disease.

### 3.7. FFR in Aortic Valve Disease

Severe aortic stenosis creates a distinct hemodynamic environment in which coronary physiology can be difficult to interpret. The combination of severe aortic stenosis and coronary artery disease behaves as two sequential stenoses [55,56]. The aortic valve acts as an upstream lesion that restricts flow into the coronary circulation, reducing the trans-stenotic pressure gradient across a downstream coronary stenosis and causing it to appear less hemodynamically significant than it truly is. This flow-limiting effect results in falsely elevated FFR values that underestimate the functional severity of the coronary lesion [57]. After aortic valve replacement, flow across the coronary stenosis increases, unmasking its true hemodynamic significance: the FFR-measured post-TAVI may be lower than the pre-TAVI for the same anatomical lesion [58]. However, this serial stenosis model is an oversimplification.

The 2026 American Heart Association (AHA) Scientific Statement describes a more complex mechanism: in severe aortic stenosis, resting coronary flow may be increased to meet the oxygen demand of the hypertrophied left ventricle, which lower non-hyperemic pressure ratios (NHPR) and may overestimate hemodynamic severity [59]. At the same time, elevated left ventricular end diastolic pressure, increased microvascular resistance, myocardial fibrosis, and capillary rarefaction all raise the FFR across a given stenosis, underestimating lesion severity [58,59,60].

The COMIC-AS study provided useful mechanistic data in this setting. In patients with severe aortic stenosis, an FFR ≤ 0.83 and an RFR ≤ 0.85 predicted myocardial ischemia more accurately before an aortic valve replacement than the conventional thresholds [61]. Six months post-valve replacement, the FFR decreased and the RFR increased. The rise in the RFR was attributed to a lower resting coronary flow after regression of the left ventricular mass and a normalization of resting hemodynamics, rather than microvascular recovery alone, even though the microvascular parameters (IMR, microvascular resistance reserve) also improved. In this cohort, 21.5% of lesions crossed the conventional FFR threshold at 6 months, with a mean FFR decrease of 0.028. Pre-TAVI FFR values in the 0.80–0.85 range may demarcate a “gray zone” that may drop below 0.80 after aortic valve replacement (TAVI/SAVR), whereas a shift to <0.75 would be less likely [58].

The FAITAVI trial addressed whether, among TAVI candidates selected for PCI, the FFR guidance was superior to angiographic guidance. A total of 320 patients were enrolled, with a median age of 86 years and a median SYNTAX score of 7 [62]. At 12 months, FFR-guided PCI was associated with significantly fewer major adverse cardiovascular and cerebrovascular events (MACCEs) compared with angiography-guided PCI (8.5% vs. 16.0%; *HR* 0.52; 95% *CI* 0.27–0.99; *p* = 0.047), driven mainly by lower all-cause mortality. The TCW trial extended this question to patients with more complex coronary anatomy, comparing FFR-guided PCI plus TAVI with the traditional surgical approach of SAVR plus CABG in patients aged ≥70 years with severe aortic stenosis and multivessel or complex coronary artery disease [63]. The trial was stopped early after enrolling 172 patients due to a significant difference favoring the percutaneous strategy. At 1 year, FFR-guided PCI plus TAVI was superior to SAVR plus CABG for the patient-oriented composite endpoint (HR 0.17, 95% CI 0.06–0.51, *p* < 0.001), with lower all-cause mortality (0% vs. 10%, *p* = 0.0025) and life-threatening bleeding (2% vs. 12%, *p* = 0.010).

Two further randomized trials addressed whether and how to revascularize coronary disease in TAVI candidates. The NOTION-3 study randomized 455 patients with severe aortic stenosis and at least one significant coronary stenosis (*FFR* ≤ 0.80 or diameter stenosis ≥ 90%) to PCI or conservative treatment alongside TAVI; FFR-guided PCI reduced the composite of death, myocardial infarction, or urgent revascularization (26% vs. 36%, *HR* 0.71, 95% *CI* 0.51–0.99) at the cost of more bleeding (*HR* 1.51) [64]. The PRO-TAVI study instead tested timing rather than indication: among 466 patients, deferral of routine PCI was non-inferior to PCI before TAVI for the primary composite clinical endpoint (*HR* 0.89, 95% *CI* 0.62–1.28, *p* < 0.001 for non-inferiority), with fewer major bleeding events in the deferral group [65]. Of note, in the PRO-TAVI trial, the FFR was not part of the mandatory protocol for determining treatment. These trials support selective, physiology- or anatomy-guided revascularization of significant lesions in TAVI candidates, while indicating that routine, non-selective PCI adds bleeding risk without clear incremental benefit.

## 4. Non-Hyperemic Pressure Ratios (NHPRs)

### 4.1. Rationale for Adenosine-Free Assessment

The FFR requires maximal hyperemia, achieved mainly via adenosine use. Although it is considered safe, shortness of breath and chest pain are common side-effects, as described above. Additionally, it increases the procedural time, costs, patient discomfort, and requires drug preparation. Consequently, despite guideline recommendations, utilization of the FFR remains lower than expected. In the ALL-RISE trial, a physiological assessment of intermediate lesions was less than 20%, suggesting that workflow rather than lack of evidence remains a major obstacle in widespread utilization of the FFR [66]. NHPRs were developed to address this gap by assessing the trans-stenotic pressure gradient without the need for a hyperemic agent.

The physiological rationale for NHPRs is based on identifying periods in the cardiac cycle where microvascular resistance is minimal and relatively stable, omitting therefore the need for administering hyperemic agents. This is most relevant during diastole, when the competing pressure waves are reduced and the coronary flow is more directly related to the pressure gradient across a stenosis [67]. Table 2 includes a comparison of all the available NHPRs.

Nonetheless, resistance during these resting periods is never as low as achieved during pharmacological hyperemia, resulting in small trans-stenotic pressure gradients and a narrower measurement range. Götberg et al. demonstrated that the whole Pd/Pa cycle has a narrower measurement range, with 62% of the values clustered around its operating point, compared to 50% for the iFR and only 35% for the FFR [68]. This is why resting indices are easier to perform but generally have less diagnostic separation than the FFR.

Using the binary thresholds 0.89 for NHPRs and 0.80 for the FFR, NHPRs agree with the FFR in approximately 80% of cases (*sensitivity of* 78.9%, *specificity of* 82.4%, *and overall accuracy of* 80.4%). Performance also varies by vessel. In an individual patient-level, meta-analysis of 2120 paired measurements, Storozhenko et al. found lower sensitivity and accuracy for NHPRs in non-LAD vessels than in the LAD *vessels* (69% vs. 87% *sensitivity,* 76% vs. 86% *accuracy*), with optimal cutoffs differing between LAD (≤0.88) and non-LAD (≤0.92) [69].

The long-term outcome data have also attracted some caution. In the 5-year pooled analysis of the DEFINE FLAIR and iFR SWEDEHEART studies, the all-cause mortality was higher with NHPR-guided management than with FFR-guided management (8.3% vs. 6.3%, *HR* 1.34, 95% *CI* 1.08–1.67) [70]. The mechanism remains uncertain, particularly because myocardial infarction and unplanned revascularization were not increased. This finding does not necessarily invalidate the NHPR, but it argues against treating it as interchangeable with the FFR in every clinical setting. Where the decision to defer a lesion subtending a large myocardial territory rests on a borderline iFR value, confirmation with the FFR is advisable until longer term individual patient data clarify whether the mortality difference is causal.

### 4.2. Instantaneous Wave-Free Ratio (iFR)

The instantaneous wave-free ratio (iFR) was the first NHPR to be widely adopted in clinical practice. Introduced by Sen et al. in 2012 [67,71], it is calculated as the ratio of distal coronary pressure to proximal aortic pressure during the “wave free period” of diastole. This period begins approximately 25% into diastole and ends 5 msec before the end of diastole. During this period, competing forward and backward pressure waves are quiescent, and microvascular resistance is at its lowest resting level, although it is never as low as during pharmacological hyperemia [72,73]. For this reason, the wave-free period is better described as a phase of relatively low and stable resistance, rather than a true equivalent of maximal hyperemia.

An iFR value ≤0.89 is generally used to define hemodynamic significance and corresponds approximately to an FFR threshold ≤0.80 [74]. This index is proprietary to Philips Healthcare and requires a compatible console. Validation studies showed approximately 80–85% agreement between the FFR and iFR, with comparable concordance against non-invasive and invasive ischemia tests, including stress echocardiography, single photon emission computed tomography (SPECT), and invasive coronary flow reserve [71].

The practical appeal of the iFR is clear. It avoids adenosine, shortens workflow, and reduces drug-related discomfort, procedural time, and cost without the need for maximal hyperemia [74]. Physiologically, the iFR and FFR are not identical. In the JUSTIFY CFR study, the iFR was more closely related to the coronary flow velocity reserve (CFVR) than the FFR, particularly in intermediate lesions. Among 216 stenoses in 185 patients, the iFR showed a stronger correlation with the CFVR than the FFR and a higher AUC for the CFVR classification (0.82 vs. 0.72, *p* < 0.001), with the closer relationship most marked in the 0.6–0.9 FFR range [75].

The clinical validation of iFR-guided revascularization came from two landmark randomized controlled trials which awarded it a class I indication in the guidelines. The DEFINE-FLAIR trial randomized 2492 patients with stable coronary artery disease to iFR-guided versus FFR-guided PCI [76]. At 1 year, iFR-guided PCI was non-inferior to FFR-guided PCI for the composite endpoint of death, myocardial infarction, or unplanned revascularization (6.8% vs. 7.0%, *p* = 0.78 for non-inferiority). The iFR-SWEDEHEART trial, enrolling 2037 patients, similarly demonstrated non-inferiority of the iFR guidance at 1 year (6.7% vs. 6.1%, *p* < 0.001 for non-inferiority) [68,77].

The clinical significance of iFR’s stronger correlation with coronary flow reserve compared to the FFR lies in its reflection of resting microvascular function. During the wave-free period, microvascular resistance is at its lowest resting level, making the trans-stenotic pressure gradient more directly related to coronary flow without the confounding effects of hyperemic agents. By contrast, the FFR measures the gradient under maximal hyperemia, which may be influenced by variable adenosine responsiveness, genetic polymorphisms in adenosine metabolism, and microvascular dysfunction that blunts the hyperemic response [75]. The stronger iFR–CFR relationship suggests that resting physiology may, in selected patients, provide a more physiologically stable assessment of stenosis severity, particularly when the hyperemic reserve is impaired. However, this does not imply superiority: it reflects a different physiological state, and the two indices are complementary rather than interchangeable [78].

Long-term follow-up data, however, have introduced important caution. At 5 years, the DEFINE-FLAIR trial showed higher mortality with iFR guidance compared to FFR guidance (HR 1.56, 95% CI 1.16–2.09), with the excess mortality confined to the revascularized arm rather than the deferred arm [79]. On the contrary, the iFR-SWEDEHEART trial showed no significant difference at 5 years (HR 1.09, 95% CI 0.84–1.43). A pooled 5-year meta-analysis of both trials by Eftekhari et al. found higher all-cause mortality with iFR-guided management (8.3% vs. 6.3%, HR 1.34, 95% CI 1.08–1.67, *p* = 0.008) [70]. The mechanism remains uncertain, particularly because the myocardial infarction and unplanned revascularization rates were not increased. Post hoc analyses suggest the excess deaths were largely from non-cardiovascular or undetermined causes, raising questions about whether the mortality signal is causally related to iFR-guided decision-making or represents a chance finding. This uncertainty does not invalidate the iFR but argues against treating it as interchangeable with the FFR in every clinical setting.

The iFR remains a useful and guideline-supported tool, with a Class I, Level A recommendation. Its results should be carefully interpreted where the resting and hyperemic physiology may diverge, including right coronary artery lesions, left main or proximal LAD disease, severe aortic stenosis, suspected microvascular dysfunction, or altered hemodynamics after contrast administration or β-blocker use [59].

### 4.3. iFR in Acute Coronary Syndromes

The iMODERN trial evaluated non-culprit lesion management after STEMI using a different physiological strategy. Nijveldt et al. randomized 1146 patients who had undergone successful primary PCI to either immediate iFR-guided PCI of non-culprit lesions during the index procedure *(n* = 558) or deferred management guided by cardiac stress MRI within 6 weeks (*n* = 588). At 3 years, iFR-guided immediate PCI was not superior to the deferred MRI-guided strategy for the composite endpoint of all-cause death, recurrent myocardial infarction, or hospitalization for heart failure (9.3% vs. 9.8%, HR 0.95, 95% CI 0.65–1.40, *p* value for superiority not significant) [80]. Both strategies were safe and effective, though the MRI-guided approach led to fewer interventions without compromising outcomes (42.6% vs. 18.7% underwent non-culprit PCI).

By contrast, FFR-guided complete revascularization has demonstrated sustained clinical benefit in the STEMI setting. The 10-year follow-up of the DANAMI-3-PRIMULTI study showed that FFR-guided complete revascularization of non-culprit lesions remained superior to treatment of the infarct-related artery alone, with a 24% reduction in the composite of all-cause death, myocardial infarction, or repeat revascularization (HR 0.76, 95% CI 0.60–0.94, *p* = 0.014), driven primarily by fewer repeat revascularizations [51]. These findings underscore that, while iFR-guided immediate intervention did not prove superior to deferred imaging-guided management, FFR-guided staged complete revascularization provides durable long-term benefit in STEMI patients with multivessel disease.

### 4.4. iFR in Aortic Stenosis

The interaction between severe aortic stenosis and resting coronary physiology presents a distinct challenge for iFR interpretation. Jo et al. studied 395 coronary lesions in 293 patients with severe aortic stenosis and demonstrated a striking divergence between the iFR and FFR in this setting. Among patients with severe aortic stenosis, the iFR classified significantly more lesions as hemodynamically significant than the FFR (66.6% with iFR ≤ 0.89 vs. 45.3% with an FFR ≤ 0.80, *p* < 0.001) [81].

This systematic overestimation by the iFR reflects the altered hemodynamic state in severe aortic stenosis. Resting coronary flow is increased to meet the elevated myocardial oxygen demand of the hypertrophied left ventricle. Because the iFR measures the trans-stenotic pressure gradient at rest, this elevated baseline flow generates a larger resting pressure drop across a given stenosis compared to what would be observed in the absence of aortic stenosis. By contrast, the FFR, which is measured during maximal hyperemia, is less influenced by the baseline flow state, as hyperemia effectively normalizes microvascular resistance across different hemodynamic conditions. Severe aortic stenosis underestimates epicardial lesion severity by the FFR by reducing the hyperemic flow, it overestimates severity by the iFR by increasing the resting flow.

In the Jo et al. study, the FFR showed a stronger association with clinical outcomes and better diagnostic concordance with stress imaging, while the iFR retained utility primarily for its high negative predictive value, a normal iFR (>0.89) reliably excluded hemodynamically significant stenosis [81]. However, an abnormal iFR in the setting of severe aortic stenosis should not be assumed to reflect true lesion-specific ischemia [82]. In patients being evaluated for TAVI, where concomitant coronary artery disease is present, confirmation with a hyperemic FFR assessment is recommended before revascularization decisions are made, particularly when the iFR suggests there is significant disease. This recommendation aligns with the findings from the COMIC-AS and FAITAVI trials.

### 4.5. Other Non-Hyperemic Indices

Following the commercial success of the iFR, several other NHPRs were developed. Most differ in how they define the period of the cardiac cycle from which the pressure signal is derived, but their overall diagnostic performance is broadly similar [83,84]. The resting full-cycle ratio (RFR, Abbott) identifies the lowest filtered mean Pd/Pa during the entire cardiac cycle, using the same threshold of an iFR ≤ 0.89. Unlike the conventional Pd/Pa, the RFR does not average pressure over the full cardiac cycle. Instead, it uses a beat-by-beat approach to identify the lowest Pd/Pa point, independent of ECG, landmark identification, and timing withing the cardiac cycle [85]. In the VALIDATE RFR [86] and REVALIDATE RFR studies, the RFR showed diagnostic equivalence to the iFR. Notably, in the VALIDATE RFR study, the lowest Pd/Pa was detected outside diastole in 12.2% of all cardiac cycles and in 32.4% of right coronary artery cycles, identifying stenoses that could be missed by indices restricted to diastole.

The diastolic hyperemia-free ratio (DFR, Boston Scientific, Marlborough, MA) uses a different approach. It calculates the average Pd/Pa over an approximated diastolic period, defined from the pressure tracing as negatively sloped segment during which instantaneous Pa is below the mean Pa. The measurement is averaged across five consecutive cardiac cycles, also using the threshold of ≤0.89. Validation studies have shown close agreement between the DFR and iFR with the DFR strongly correlating with the FFR [87].

Resting Pd/Pa is the simplest non-hyperemic index. It averages the distal-to-proximal pressure ratio across the entire cardiac cycle and uses a threshold of ≤0.91. Its main limitation is lower diagnostic resolution. This is better understood as a signal-to-noise issue rather than as a simple effect of systolic compression. Because the majority of coronary flow occurs in diastole, the systolic portion of the signal contributes less useful information. Including it narrows the dynamic range of Pd/Pa compared with the iFR. Lee et al. demonstrated that the iFR changed more clearly than Pd/Pa with increasing stenosis severity across angiographic measures, basal and hyperemic stenosis resistance, and hyperemic myocardial blood flow [85]. Götberg et al. showed that 62% of the Pd/Pa values cluster around its operating point versus 50% for the iFR, resulting in lower discriminatory power [68]. Resting Pd/Pa is therefore easy to obtain but is more vulnerable to drift and less suited for a detailed pullback assessment in serial or diffuse disease.

A pullback-based assessment of disease pattern has been extended beyond a hyperemic FFR. The iFR pullback with pressure co-registration is used to localize pressure loss and to plan virtual PCI, and a pullback pressure gradient index has been applied to resting and angiography-derived indices, including a Murray’s law-based quantitative flow ratio pullback pressure gradient index (µFR-PPGi) [88]. The narrower dynamic range of resting indices nonetheless limits detailed pullback discrimination, and the resting pullback behavior can be less predictable than hyperemic pullback: in patients undergoing TAVI, the FFR decreased predictably in vessels with major focal gradients, whereas the iFR changes were unpredictable. The pullback pressure gradient is therefore most robustly established with a hyperemic FFR; while resting and wire-free pullback remain useful for lesion localization and procedural planning [89].

### 4.6. Technical Considerations for NHPR Use

Several technical issues should be taken into account when NHPRs are used. Since resting trans-stenotic pressure gradients are smaller than those achieved by hyperemic gradients, NHPRs are more vulnerable to pressure drift and signal noise than the FFR. A drift of 2–3 mmHg may have little effect when the hyperemic gradient is 15–20 mmHg, but it may be of clinical importance when the resting gradient is only 3–5 mmHg [84]. Piezoelectric sensors drift more than optical sensors and ±0.02 is considered an acceptable drift threshold, a magnitude proportionally far more consequential for the NHPR than the FFR [59].

Heart rate can also affect the NHPR interpretation. Hennigan et al. identified the baseline heart rate as an independent determinant of NHPR and FFR discordance on multivariable analysis [34]. Tachycardia shortens diastole, reducing the sample window for diastolic indices such as the iFR and DFR. For whole cycle indices, such as the RFR and resting Pd/Pa, the measurement window remains available but the relative contribution of diastole to the full cycle signal is reduced [69].

β-Blockers may also influence resting physiological indices. In a study of 197 patients with 223 lesions, Verdoia et al. found that preprocedural β-blocker use was associated with numerically higher iFR values (0.94 ± 0.06 vs. 0.92 ± 0.06, *p* = 0.11) and a significantly lower rate of positive iFR (14.9% vs. 27.5%, *p* = 0.04). On multivariable analysis, β-blocker therapy was an independent predictor of iFR classification (OR 0.48, 95% *CI* 0.23–0.98, *p =* 0.05), translating into approximately half the odds of a positive iFR [90]. The mechanism is likely more complex than heart rate reduction alone. Different β-blocker generations may have different effects on coronary flow reserve through interactions between reduced resting flow, variable hyperemic flow, and minimal coronary resistance, while vasodilating agents, such as carvedilol and nebivolol, may improve hyperemic flow through a-blockade or nitric oxide mediated effects [91]. β-blocker use may reduce resting pressure gradients and contribute to false-negative NHPR results in selected patients.

Beta-blocker intake, a category of agents prescribed in a large proportion of patients with coronary disease, approximately halves the odds of a positive iFR. Therefore, a borderline negative non-hyperemic result in a patient on β-blockade with a high pretest probability of ischemia should not be regarded as definitive [90]. Confirmation with the FFR, or an interpretation in the light of this bias, is preferable to ignoring the effect.

## 5. Discordance Between FFR and NHPRs

Discordance between the FFR and iFR occurs in approximately 20% of lesions, typically near the diagnostic thresholds [35,92]. The FIGARO study provided the two main patterns of discordance: FFR-negative and iFR-positive lesions, that were defined as an FFR > 0.80 with an iFR ≤ 0.89 and FFR-positive and iFR-negative lesions, defined as an FFR ≤ 0.80 with an iFR > 0.89 [35]. These patterns reflect the fact that hyperemic and resting indices interrogate the coronary physiology under different flow conditions.

In the FFR-negative/iFR-positive pattern, the impaired vasodilatory reserve may blunt the hyperemic response: reduced flow augmentation during hyperemia attenuates the trans-stenotic gradient, producing a falsely normal FFR, while the resting iFR correctly identifies the lesion as hemodynamically significant. Lee et al. confirmed this interpretation, demonstrating that the low-iFR/high-FFR group had coronary flow reserve and resistance reserve ratio values comparable to the concordantly abnormal group, indicating impaired vasodilatory capacity [85]. This pattern is associated with smoking, chronic kidney disease, increased microvascular resistance, as well as polymorphisms affecting endothelial nitric oxide synthase and heme oxygenase pathways [35].

The FFR-positive/iFR-negative pattern reflects an augmented vasodilatory response to adenosine, in which high coronary flow during hyperemia generates a large trans-stenotic gradient even when the lesion is not flow-limiting at rest. Lesions in this category often demonstrate a preserved CFR; however, a preserved CFR should not be considered fully reassuring, as the data from the DEFINE-FLOW registry and the subanalysis of the COMPARE-ACUTE study indicate that an abnormal FFR retains independent prognostic significance for target vessel failure even in the presence of a preserved CFR [93,94].

The clinical consequences of this pattern are most relevant when the affected vessel is the left main or proximal LAD. In these territories, the large perfused myocardial mass amplifies the hyperemic flow response to adenosine, generating a proportionally larger trans-stenotic gradient that renders the FFR positive while the resting iFR remains above 0.89. Kobayashi et al. examined this territory-specific effect in the CONTRAST study and underlined the reduced diagnostic accuracy of adenosine-free indices in LM/proximal LAD [95]. Clinically, the more consequential error in this territory is not a false-positive iFR, which would prompt revascularization, but a false-negative iFR that reassures the operator while the FFR would have been positive. Deferral of a left main or proximal LAD stenosis on the basis of a reassuring iFR carries consequences disproportionate to those of the same error in vessel subtending smaller myocardial territory. When an iFR assessment of the left main or proximal LAD yields values at or near the grey zone, an FFR should be performed before any decision to defer.

### 5.1. Influence of Lesion Location

The diagnostic performance of NHPRs varies by coronary territory. In left main and proximal LAD lesions, the basis for discordance is the large myocardial territory supplied. During adenosine-induced hyperemia, the increase in coronary flow is greater in vessels subtending a larger myocardial mass, which in turn produces a larger trans-stenotic gradient that is captured by the FFR but may be underestimated by resting indices. This explains why the FFR-positive/iFR-negative pattern clusters in these territories. The FIGARO study associated this pattern with the right coronary artery [35], while the CONTRAST study identified the left main and proximal LAD [92,95]. The clinical relevance of this concern is that functionally significant proximal lesions in vessels supplying large amounts of myocardium carry the worst prognosis, and accurate classification is therefore critical.

The right coronary artery presents a different challenge. The RCA is subject to lower systolic compression than the LCA because right ventricular wall stress is substantially lower than left ventricular wall stress. As a consequence, the backward decompression wave, the diastolic suction force generated by myocardial re-expansion, is smaller in the RCA, and peak coronary flow occurs relatively later, in late systole and early diastole rather than in mid-diastole, as in the LCA. This means the RCA is diastolic dominant but less markedly so, with a broader, flatter flow velocity profile across the cardiac cycle compared with the left system. Earlier Doppler studies raised concern that diastolic-only indices may therefore not perform well in this vessel. The IDEAL study analyzed 482 simultaneous pressure and flow measurements and clarified that true systolic flow predominance is rare in both systems, occurring in only 2.1% of cases [96]. However, the RCA diastolic-to-systolic velocity ratio was significantly lower than the LCA (1.53 vs. 1.85 at rest, *p* < 0.001) [96], confirming that the physiological basis for the concern is real, even if frank systolic predominance is uncommon. The wave-free diastolic window samples a less representative portion of the total RCA flow than it does in the left system, and microvascular resistance during this period may be less uniformly stable. This mechanistic difference translates into measurable diagnostic degradation. The VALIDATE RFR study confirmed that both RFR and iFR sensitivity against the FFR was lower in the right coronary artery, with the RFR identifying the lowest Pd/Pa outside diastole in 32.4% of right coronary artery cardiac cycles [86]. Switching to whole cycle indices does not resolve the problem: the individual patient level meta-analysis by Storozhenko et al. showed lower diagnostic performance of NHPRs in non-LAD vessels compared with LAD lesions for sensitivity (69% vs. 87%) and accuracy (76% vs. 86%), regardless of whether the index was diastolic-only or whole cycle [69].

### 5.2. Impact of Aortic Valve Disease on Discordance

The interaction between severe aortic stenosis and coronary physiology was studied by Jo et al. [81]. There was a striking divergence between the iFR and FFR in this setting. Among patients with severe aortic stenosis, the iFR classified more lesions as hemodynamically significant than the FFR (66.6% *with iFR* ≤ 0.89 vs. 45.3% *with FFR* ≤ 0.80, *p* < 0.001). Severe aortic stenosis affects resting and hyperemic indices differently. The iFR may overestimate lesion severity because the resting coronary flow is increased in response to left ventricular hypertrophy and higher myocardial oxygen demand. The FFR appears to be less affected by aortic stenosis severity and more closely associated with prognosis in this patient cohort. The iFR retained utility for the exclusion of significant stenosis, given its high negative predictive value, but the iFR in severe aortic stenosis patients should be interpreted with caution. All in all, in patients being evaluated for TAVI where concomitant CAD is present, confirmation with a hyperemic assessment before revascularization decisions are made is required.

## 6. Virtual Hemodynamic Assessment of Coronary Lesions

### 6.1. Principles of Angiography-Derived Physiology

Angiography-derived physiology aims to estimate the functional significance of a coronary stenosis without a pressure wire or pharmacological hyperemia. Anatomical imaging from invasive coronary angiography, computed tomography coronary angiography (CTCA), or intravascular imaging (IVUS, OCT) is used to 3D-reconstruct the coronary vessel and computational fluid dynamics (CFD) modeling estimate of trans-stenotic pressure gradients [97,98].

Simplified analytical models apply the Bernoulli and Poiseuille principles to estimate pressure loss across stenotic segments, and are typically used by angiography-derived systems. An FFR-CT uses more complex computational fluid dynamics based on Navier–Stokes CFD [99]. Hyperemic flow is not measured directly in these systems; it is estimated by correlations between vessel size and myocardial territory or, in the case of the quantitative flow ratio (QFR), through a frame count analysis of contrast opacification [100,101].

Diagnostic accuracy depends on image quality, reconstruction method, flow estimation, operator training, and the specific algorithm used. Randomized control trials have contrasting results. In the FAVOR III Europe study, the QFR did not demonstrate non-inferiority over the FFR [102]; whereas, in the FAST III [103] and ALL RISE [104] studies, vessel FFR and FFRAngio met non-inferiority against pressure wire-based strategies at 1-year.

### 6.2. Quantitative Flow Ratio (QFR)

The QFR estimates the FFR from a standard coronary angiography without the need for a pressure wire or pharmacological hyperemia. It combines 3D quantitative coronary analysis with simplified dynamic modeling, and the flow velocity is estimated from the frame count analysis of contrast opacification [105]. As with the FFR, a value of the QFR ≤ 0.80 defines hemodynamic significance.

An accurate QFR analysis depends heavily on image quality and acquisition technique. An accurate analysis requires at least two angiographic projections, ideally separated by ≥25 degrees, without vessel overlap or foreshortening, table panning, or inadequate contrast opacification. Long and brisk contrast injections are important, since poor opacification can affect the frame count analysis and vessel reconstruction. Failure to meet these requirements leads to inaccurate 3D reconstruction and unreliable QFR calculations [97].

The clinical evidence for the QFR is mixed. The FAVOR III China study randomized 3825 patients with at least one intermediate stenosis (50–90% diameter stenosis) to QFR-guided versus angiography-guided PCI [106]. At 1-year, QFR guidance reduced MACE (5.8% vs. 8.8%, *HR* 0.65, 95% *CI* 0.51–0.83, *p* = 0.0004). At 2-years, MACE was 8.5% vs. 12.5% (*HR* 0.66, 95% *CI* 0.54–0.81, *p* < 0.0001), with myocardial infarction HR 0.58 (*p* = 0.0002) and ischemia-driven revascularization HR 0.71 (*p* = 0.02) [107]. The benefit was greatest when the QFR altered the revascularization strategy compared to invasive angiography alone.

The FAVOR III Europe study reached a different conclusion. The QFR did not demonstrate non-inferiority to the FFR for guiding revascularization in patients with intermediate coronary stenoses [102]. Demonstrating superiority over angiography alone is not the same as replacing invasive pressure wire-based physiology. The former shows that physiology improves visual decision-making and the latter requires enough accuracy and reproducibility to match the FFR.

The REPEAT-QFR substudy highlighted why this may be difficult in practice. Agreement between in-procedure QFR and core-laboratory QFR was only 72%, with a Spearman correlation of 0.58 and wide limits of agreement (−0.26 *to* 0.29) [108]. Variability was greater with suboptimal angiographic quality, poor in-procedure analysis quality, high SYNTAX score, and diabetes. A subsequent post hoc analysis of the FAVOR III Europe study found that QFR-based deferral was associated with higher MACE than FFR-based deferral among patients with a complete study lesion deferral (5.6% vs. 2.8%, *adjusted HR* 2.07, 95% *CI* 1.07–4.03, *p* = 0.03) [109], suggesting that inappropriate deferral was driven in part by false-negative QFR results.

### 6.3. FFRAngio and vFFR

Recent randomized evidence has been favorable for angiography-derived physiology, although from different computational platforms. The FAST III trial randomized 2211 patients with intermediate coronary lesions to vessel FFR (vFFR)-guided versus FFR-guided revascularization [103]. At 1 year, vFFR guidance was non-inferior to FFR guidance for the composite of death, myocardial infarction, or revascularization (*event rates:* 7.5% vs. 7.5%, *risk difference* −0.02 *percentage points,* 95% *CI* −2.25 *to* 2.21, *p* = 0.004 *for non-inferiority*).

The ALL-RISE study evaluated a different angiography-derived platform. FFRAngio (CathWorks Ltd., Kfar Saba, Israel) was non-inferior to a pressure wire-guided strategy for the 1-year composite of all-cause death, myocardial infarction, and unplanned clinically-indicated coronary revascularization (6.9% vs. 7.1%, *p* < 0.001 *for non-inferiority*) [66]. FFRAngio estimates lesion physiology from a standard coronary angiography using a cloud-based CFD platform to compute the FFR, automatically reconstructing the entire coronary tree from a single angiographic run.

The divergent results across angiography-derived platforms reflect concrete methodological differences rather than a failure of the wire-free indices. Primarily, the trials asked different questions against different comparators: the FAVOR III Europe study tested the QFR for non-inferiority against wire-based FFR, whereas the FAST III study compared vessel FFR with the FFR and the ALL-RISE study compared FFRAngio with a pressure wire strategy, each with its own endpoint and population.

Moreover, the platforms differ computationally: the QFR derives flow from a two projection frame count analysis and a 3D quantitative coronary analysis, which is sensitive to projection selection, vessel overlap, foreshortening, and injection quality. The REPEAT-QFR substudy found only 72% agreement between the in-procedure and core-laboratory QFR, with greater variability in complex anatomy, high SYNTAX score, and diabetes [108]. FFRAngio reconstructs the entire coronary tree from multiple runs using a cloud-based CFD model, and vessel FFR combines a 3D quantitative coronary analysis with computational modeling, approaches less dependent on a single projection pair. Operator workflow and the online-versus-core laboratory analysis differed between trials. These factors explain why one platform failed non-inferiority while others succeeded, and reinforce that angiography-derived indices should be adopted platform by platform rather than as a single class.

Implementation barriers also shape real-world performance. Angiography-derived indices require standardized, high quality image acquisition, with at least two well-separated projections, minimal overlap and foreshortening, and adequate contrast opacification. They are subject to a learning curve and depend on operator training, and the gap between the core-laboratory and in-procedure online analysis demonstrated in the REPEAT-QFR study shows that reproducibility in routine practice, rather than the underlying algorithm, is often the limiting factor. Software cost, licensing, and integration into the cath laboratory workflow further influence uptake.

### 6.4. CT-Derived FFR

FFR derived from computed tomography angiography (CT-FFR), allows for a non-invasive functional assessment before entering a cardiac catheterization laboratory. It is most reliable when the CCTA image quality is high, and requires appropriate heart rate control (≤60 *bpm*), the use of nitroglycerin and β-blockers when appropriate, and acquisition protocols that minimize motion and misalignment and inclusion of the entire coronary tree [110].

FFR-CT has been validated in the DISCOVER FLOW [111], DeFACTO [112], and NXT [113] studies, while the PLATFORM study [114] showed its potential role as a gatekeeper to invasive coronary angiography. Patients with an FFR-CT > 0.80 can avoid invasive procedures, while those with an FFR-CT ≤ 0.80 warrant catheterization.

Several limitations apply. Diagnostic accuracy is lower near the diagnostic threshold and in heavily calcified vessels. Blooming artifacts impair a lumen assessment [115]. The image rejection rate varies between 2.9% and 13% across studies, limiting the workflow [116]. A non-significant but consistent signal of higher myocardial infarction rates in some FFR CT-guided strategies has been observed [117]. The FFR should not be used as a rigid binary test at the margins. Values close to 0.80 are best interpreted alongside symptoms, plaque burden, stenosis location, CCTA quality, and expected clinical consequences of invasive angiography.

### 6.5. Current Guideline Recommendations for Virtual Coronary Physiology

The 2024 ESC Guidelines on Chronic Coronary Syndromes provide a Class I, Level B recommendation for the QFR (significant ≤ 0.80) for the functional assessment of intermediate diameter stenoses during invasive coronary angiography [5]. As of the 2024 guideline publication, the QFR recommendation preceded the negative FAVOR III Europe result and, together with the confirmatory FAST III and ALL-RISE data for other platforms, guideline updates are anticipated. The SCAI Roundtable emphasized that findings from one ADP platform should not be generalized to others, given the heterogeneous evidence across systems [118].

## 7. Assessment of Coronary Microcirculation

A substantial portion of patients, up to 40%, with angina and positive non-invasive stress tests referred for coronary angiography have non-obstructed coronary arteries [119]. This population has been mislabeled as having non-cardiac chest pain and reassured without further evaluation, yet this designation is not benign. Recent data have reported a nearly fourfold increase in the incidence of mortality and MACE in ischemia with non-obstructed coronary artery (INOCA) patients compared with those without the diagnosis [120], with the highest risk concentrated in those with microvascular angina and impaired coronary flow reserve. Women account for 50–70% of INOCA referrals, while the condition also affects 30–50% of men investigated for angina [121,122].

Patients with INOCA and angina with non-obstructive coronary arteries (ANOCA) describe clinical syndromes with several underlying pathophysiological mechanisms, including coronary microvascular dysfunction (CMD), epicardial vasospastic angina (VSA), and their combination [6,7]. These mechanisms exist solely or in combination with obstructive epicardial disease, complicating both diagnosis and management. CMD frequently accompanies obstructive CAD. In the CE-MARC 2 substudy, 39% of patients with obstructive CAD had an abnormal index of microcirculatory resistance, and 53% had abnormal CFR [123].

### 7.1. Coronary Flow Reserve (CFR)

The coronary flow reserve (CFR) is the ratio of hyperemic to resting coronary blood flow. It reflects the vasodilatory capacity of coronary circulation as a whole, incorporating both epicardial and microvascular components. The CFR can be measured invasively using Doppler Flow velocity using a ComboWire or FloWire or with thermodilution using a pressure and temperature sensor wire [124]. The optimal CFR threshold depends on the measurement modality. The thermodilution threshold of 2.0 has poor sensitivity and Demir et al. recommended a threshold of 2.5 regardless of modality [125].

This threshold heterogeneity is compounded by measurement modality. Doppler-derived coronary flow velocity reserve and thermodilution-derived CFR are not interchangeable, bolus thermodilution is limited by the reproducibility of manual injections, and continuous thermodilution improves reproducibility [126,127]. A single universal cutoff, commonly 2.0, with 2.5 proposed by some, should therefore be applied with awareness of the technique used and between center variation. Consistent with this, microvascular classification can differ substantially between methods: using microvascular resistance reserve, agreement between Doppler echocardiography and bolus thermodilution was poor (κ ≈ 0.08), with microvascular dysfunction diagnosed in 20.6% versus 32.8% of patients depending on modality [128].

A CFR < 2.0–2.5 indicates impaired vasodilatory capacity and is associated with worse prognosis and persistent symptoms, independent of the presence of epicardial stenosis. When the FFR or iFR is normal, a reduced CFR points towards microvascular dysfunction. The CFR can be reduced in patients with a normal FFR, identifying a population at increased cardiovascular risk that would not be detected by an epicardial pressure assessment alone. The FFR and CFR discordance is observed in up to 30–40% of vessels, making it a common scenario [39,129].

The DEFINE-FLOW registry revealed that patients with an FFR ≤ 0.80 but a CFR > 2.0 with deferred PCI had comparable outcomes to those who underwent revascularization, suggesting that the CFR may identify patients who can safely defer PCI even with an abnormal FFR [130]. On the contrary, a substudy of the randomized COMPARE-ACUTE trial evaluated patients with coronary lesions showing a positive FFR but a preserved pressure-bounded CFR, and compared PCI with medical therapy. Patients with FFR-positive coronary lesions but a preserved CFR had more clinical events when treated medically vs. those treated with PCI. An abnormal FFR does not always reflect the same physiological risk when the coronary flow reserve is preserved, reinforcing the need to interpret pressure-derived indices together with a flow-based assessment in selected patients [94].

### 7.2. Index of Microcirculatory Resistance

The index of microcirculatory resistance (IMR) provides a quantitative measure of minimal microvascular resistance during maximal hyperemia. It is calculated as distal coronary pressure multiplied by mean transit time, *IMR* = *Pd* × *Tmn at hyperemia* [131].

Compared with the CFR, the IMR is less influenced by resting flow conditions and is more specific to microcirculation. The IMR is independent of resting hemodynamics and myocardial mass, although mean transit time measurement has some form of operator dependence and positional variability [132].

An IMR value > 25 is the most commonly used threshold for abnormal microvascular function and is supported by contemporary guidelines [59]. The optimal cutoff may vary according to patient population and clinical setting. In patients with INOCA, one study found that an IMR threshold > 18 provided the best prognostic discrimination for MACE [133]. Doppler-derived hyperemic microvascular resistance (HMR) provides a related measure using flow velocity rather than thermodilution, with abnormal thresholds generally reported between >1.9 and 2.5 mmHg/cm/s, and >2.5 used in some studies. The Coronary Microvascular Disease Registry reported that 25.3% of symptomatic patients with non-obstructive CAD had confirmed microvascular dysfunction, with a mean IMR of 36.26 ± 19.23 [134].

### 7.3. Vasospasm Provocation Testing

Coronary vasospasm may involve the epicardial arteries, microcirculation, or both. It is a functional disorder in which excessive vasoconstriction leads to myocardial ischemia despite the absence of a fixed flow limiting stenosis. Provocative testing with intracoronary acetylcholine (ACh) is the reference test for diagnosis [135]. The COVADIS (Coronary Vasomotion Disorders International Study Group) criteria, define a positive test for epicardial spasm as transient total or subtotal artery narrowing >90% constriction, with reproduction of the patient’s usual symptoms and ischemic ECG changes [136].

Microvascular spasm is diagnosed when symptoms and ischemic ECG changes occur without significant epicardial obstruction, usually <90% diameter reduction, reflecting vasoconstriction at the pre-arteriolar level (vessels < 500 μm) [137]. At the epicardial level, endothelial dysfunction is suggested by paradoxical vasoconstriction in response to ACh (≥20% or >0% depending on institutional criteria) and, at the microvascular level, an inadequate increase in coronary blood flow, commonly <50% in response to Ach, supports endothelium-dependent vasodilation [138]. Low-dose ACh (2–20 μg intracoronary) tests endothelium-dependent vasodilation, while high-dose ACh (100–200 μg) provokes vasoconstriction through direct smooth muscle effects. Incremental dosing allows differentiation between endothelial dysfunction, microvascular spasm, and epicardial spasm [139].

Several technical considerations should be considered. Nitroglycerin is routinely administered prior to a physiological assessment but may affect the outcome of subsequent vasoconstriction testing depending on the interval between tests, by blunting the Ach response [140]. A Doppler flow measurement is preferred for vasospasm testing because it allows for the continuous monitoring of flow during the provocative challenge [141]. The choice of spasm provoking agent, dose, duration, and target vessel selection vary between centers.

### 7.4. The CorMicA Paradigm

The CorMicA (Coronary Microvascular Angina) trial was the landmark randomized trial that established the practical value of invasive coronary function testing in patients with ANOCA. A total of 151 patients were enrolled and randomized to either invasive coronary function testing (CFR, IMR, Ach provocation) with linked stratified medical therapy or standard angiography-guided care [9].

Treatable endotypes were identified in most patients, including microvascular angina (abnormal CFR and/or IMR), vasospastic angina (positive Ach testing), and combined disorders. Treatment was then tailored to the identified mechanism. At 1 year, patients in the intervention group experienced significantly greater improvement in angina symptoms (22–27% improvement in Seattle Angina Questionnaire scores) and quality of life, with sustained benefits at follow-up [142].

The subsequent ILIAS ANOCA trial strengthened the evidence. A total of 153 patients were randomized, without obstructive CAD who had undergone coronary function testing to disclosure of results combined with tailored therapy versus blinded standard care and found that the intervention group had significantly improved Seattle Angina Questionnaire summary scores at 6 months (intervention effect 9.4 units, 95% CI 3.9 to 14.9, *p* = 0.001) [143].

Invasive coronary function testing may identify treatable mechanisms, provide prognostic information when the CFR or IMR is abnormal, and prevent empiric treatment choices that may be inappropriate, such as β-blocker use in vasospastic angina [144]. Most importantly, however, it provides the patients with an explanation for their symptoms that were previously dismissed due to normal coronary arteries.

## 8. Integrating Physiological Assessment in Clinical Practice

Management of suspected or established CAD is strongest when the anatomical findings are interpreted alongside the physiological data. No single index describes the entirety of the coronary circulation.

**Figure 1:**
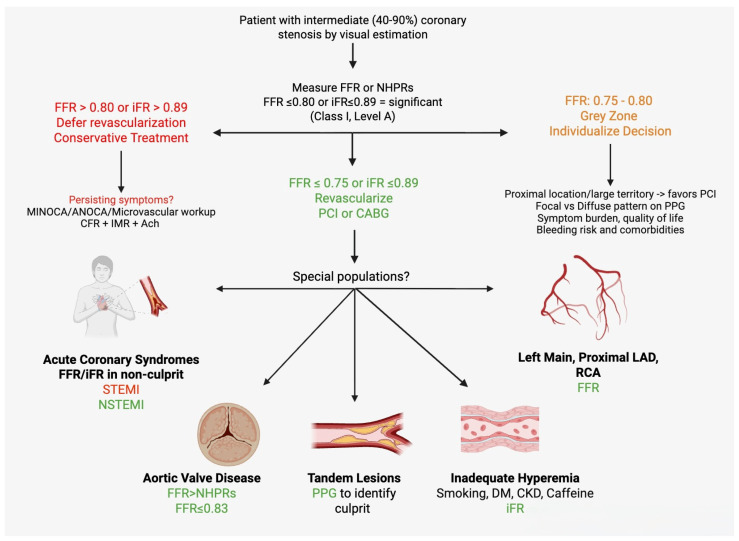
A practical algorithm for physiological assessment of intermediate coronary stenoses.

The FFR and NHPR address whether epicardial lesions cause hemodynamically significant obstruction. The CFR evaluates the vasodilatory reserve of the coronary circulation as a whole, including both epicardial and microvascular components. The IMR complements the CFR by providing a more direct estimate of minimal microvascular resistance and is less dependent on resting flow, even though it relies on transit time as a surrogate for flow. Ach testing assesses vasomotor reactivity and tendency toward epicardial or microvascular spasm. Figure 1 synthesizes a practical algorithm for the physiological assessment of intermediate coronary stenoses, and Table 3 has a summary of the landmark randomized trials and key registries in intracoronary physiological assessment.

## 9. Future Directions

Several questions remain unresolved. Primarily, longer term patient level analyses are required to clarify if the mortality benefit in the pooled iFR vs. FFR data is a true treatment-related difference. Moreover, angiography-derived physiology requires more direct platform to platform evaluation. Trials such as the FAVOR III Europe, FAST III and ALL-RISE suggest that wire-free indices should not be treated as a single class and comparative studies in similar patient populations would help define where each technology performs best. After all, wireless modalities might serve as a gate-keeper before entering the cath lab.

Automation is another important direction. Integration of artificial intelligence (AI) and machine learning into coronary angiography may improve vessel reconstruction, reduce operator dependence, and make a physiological assessment easier to use in routine practice. These tools will require rigorous validation, transparent algorithms, and randomized controlled trials to prove they can provide benefit in clinical decision making rather than simply producing faster measurements [145].

Clear pathways are required for integrating invasive and non-invasive physiology. FFR-CT, stress imaging, angiography-derived indices, pressure wire assessment, CFR, IMR, and acetylcholine testing should not compete as isolated tests, but we need to define when each test should be used, in which patient, and when, during the diagnostic pathway.

## 10. Conclusions

Coronary physiology has become an important part of contemporary CAD assessment. Evidence from the DEFER and FAME program, as well as subsequent randomized and registry studies have shown that a physiological lesion assessment can improve patient selection for revascularization and reduce unnecessary interventions. The FFR remains the most extensively validated invasive physiological index. Its value lies not only in the conventional threshold of ≤0.80, but in the continuous relationship between the FFR values, ischemic burden, and clinical risk.

NHPRs have expanded the practical use of physiology-guided decision-making by avoiding adenosine and simplifying workflow. Their 1-year non-inferiority to the FFR supported their incorporation into guideline recommendations. Longer-term data, however, have introduced uncertainty, particularly the mortality signal observed in pooled trial analyses. This signal has not been fully mechanistically explained and has not been consistently reproduced in registry-level data. Until longer-term data are provided, the FFR should remain the preferred confirmatory tool in settings where resting and hyperemic physiology are more likely to diverge, including proximal large territory vessels, severe aortic valve disease, suspected microvascular dysfunction, and altered resting hemodynamics.

Angiography-derived, wire-free physiology has now entered a more demanding phase of evaluation. The divergent results of the FAVOR III Europe, FAST III, and ALL RISE studies show that these technologies should not be judged as a single class. Each platform relies on different computational assumptions, image acquisition requirements, and validation data. Their potential is considerable, since they might serve as gate-keepers before referring a patient for invasive procedures.

A major recent advance has been the recognition that coronary physiology extends beyond the epicardial vessel. Invasive coronary function testing can identify microvascular and vasospastic endotypes in patients with ANOCA or INOCA, allowing treatment to be directed toward the underlying mechanism rather than the angiographic appearance alone. Trials such as CorMicA and ILIAS ANOCA have shown that this approach can improve symptoms and quality of life, offering a more coherent diagnostic and therapeutic pathway for patients who were previously reassured or dismissed after a “normal” angiogram.

The central challenge now is integration. No single measurement captures the entire circulation. The role of the interventional cardiologist is to select the appropriate tool for the clinical question, recognize when combined assessment is needed, and interpret discordant results in context. Coronary physiology should not replace clinical judgment or anatomical assessment, but it should refine both. The future of coronary intervention will depend less on treating what appears severe on angiography and more on identifying which abnormalities are physiologically meaningful, clinically relevant, and modifiable.

## Figures and Tables

**Table 1:** Adverse events related to adenosine administration during fractional flow reserve measurement [32].

Side Effect	Cited Rate	Meta-Analysis Rate (95% CI)	Route Most Associated
Chest pain	35%	29% (2–91%)	Mostly IV
Dyspnea	20%	20%	Mostly IV
Arrhythmia	3.3%	3% (1–16%)	IC Bolus
Nausea	0.9%	0.9%	Mostly IV
Bronchospasm	0.2%	0.2% (0.0–0.7%)	IV and IC, avoid in asthma
Vessel injury	0.4%	0.5% (dissection 0.2%, occlusion 0.2%, perforation 0.1%)	Procedure-related, route-independent
Hypotension	0.9%	0.8% (0.3–2.1%)	Mostly IV

Cited rates from registries and pooled rates from a contemporary meta-analysis. The cited rates fall within the confidence intervals of the meta-analysis, although the wide intervals for chest pain (2–91%) and arrhythmia (1–16%) reflect substantial heterogeneity across the studies. IC: intracoronary, IV: intravenous.

**Table 2:** Comparison of currently available non-hyperemic pressure ratios (NHPRs).

Index	Calculation	Cardiac Cycle	Manufacturer	Threshold	Key Limitation	Clinical Evidence
iFR	Pd/Pa during the wave-free period of diastole	Wave-free period (diastole)	Philips	≤0.89	Lower specificity in LM/proximal LAD; Lower sensitivity in non-LAD Unresolved 5-year mortality signal	Validated by DEFINE-FLAIR and iFR-SWEDEHEART (non-inferior to FFR at 1 year). A 5-year pooled meta-analysis showed higher all-cause mortality (HR 1.34); DEFINE-FLAIR
RFR	Lowest filtered mean Pd/Pa over the entire cardiac cycle, unbiased beat-by-beat	Whole cycle	Abbott	≤0.89	Captures nadir outside diastole; Limited long-term outcome data vs. iFR	VALIDATE RFR and RE-VALIDATE RFR: diagnostic equivalence to iFR. Captures lowest Pd/Pa outside diastole in 12% of cycles overall and 32% of RCA cycles.
DFR	Average Pd/Pa over the approximated diastolic period across 5 consecutive cycles	Diastole	Boston Scientific	≤0.89	Diastolic-only window narrows with tachycardia.	Diagnostically equivalent to iFR in multiple validation studies.
Pd/Pa (resting)	Mean Pd/Pa over the entire cardiac cycle at rest	Whole cycle	Non-proprietary	≤0.91	Lower diagnostic resolution and higher susceptibility to drift.	Excellent agreement with iFR and FFR; narrowest dynamic range (62% of values cluster around the operating point)

DFR, diastolic hyperemia-free ratio; FFR, fractional flow reserve; iFR, instantaneous wave-free ratio; LAD, left anterior descending artery; LM, left main; Pd/Pa, ratio of mean distal to proximal coronary pressure; RCA, right coronary artery; RFR, resting full-cycle ratio.

**Table 3:** Summary of key clinical trials in coronary physiology-guided revascularization.

Trial	N	Design	Comparison	FU	Primary Endpoint	Key Result
DEFER	325	RCT	Defer vs. PCI if FFR > 0.75	15 year	MACE	Deferral safe; no excess death/MI at 15 yr
FAME I	1005	RCT	FFR-guided vs. angio-guided PCI (multivessel)	1 year	Death, MI, repeat revasc.	FFR-guided 13.2% vs. 18.3% (*p* = 0.02); fewer stents, lower cost
FAME 2	1220 (888 rand.)	RCT	PCI + OMT vs. OMT alone (FFR ≤ 0.80)	5 years	Death, MI, urgent revasc.	PCI 13.9% vs. 27.0% (HR 0.46, *p* < 0.001); stopped early
FAME 3	1500	RCT	FFR-guided PCI vs. CABG (3-vessel)	1 years	Death, MI, stroke, revasc.	Did not meet non-inferiority vs. CABG (HR 1.50). 5-yr: no significant difference (HR 1.16, 0.89–1.52)
Zimmermann pooled	~2400	Pooled (patient-level)	PCI vs. OMT for FFR ≤ 0.80	3 years	Cardiac death or MI	PCI reduced cardiac death/MI: HR 0.72 (0.54–0.96, *p* = 0.023)
IRIS-FFR	5846	Observational registry	FFR-guided treatment, real-world	2 years	MACE	Continuous FFR–MACE relationship (HR 1.06 per 0.01 decrease)
DEFINE-FLAIR	2492	RCT	iFR-guided vs. FFR-guided PCI	5 years	Death, MI, unplanned revasc.	1-yr non-inferior (6.8% vs. 7.0%); 5-yr ↑ mortality with iFR (HR 1.56, 1.16–2.09)
iFR-SWEDEHEART	2037	RCT	iFR-guided vs. FFR-guided PCI	5 years	Death, MI, unplanned revasc.	Non-inferior at 1 and 5 yr (5-yr HR 1.09)
Eftekhari pooled	4511	Pooled (patient-level)	iFR vs. FFR	5 years	All-cause mortality, MACE	↑ mortality with iFR (HR 1.34, 1.08–1.67); undetermined causes
SWEDEHEART registry	~24,000	Observational registry	iFR vs. FFR (propensity-matched)	5 years	MACE, all-cause mortality	No significant difference (adjusted HR ~0.96)
FAVOR III China	3825	RCT	QFR-guided vs. angio-guided PCI	2 years	MACE	1-yr 5.8% vs. 8.8% (HR 0.65); 2-yr 8.5% vs. 12.5% (HR 0.66)
FAVOR III Europe	2000	RCT	QFR-guided vs. FFR-guided PCI	1 year	Death, MI, revasc.	QFR failed non-inferiority vs. FFR
FAST III	2211	RCT	vFFR-guided vs. FFR-guided PCI	1 year	Death, MI, revasc.	Non-inferior: 7.5% vs. 7.5% (*p* = 0.004 for NI)
ALL-RISE	1930	RCT	FFRangio vs. pressure wire	1 year	Death, MI, revasc.	Non-inferior: 6.9% vs. 7.1% (*p* < 0.001 for NI)
iMODERN	1146	RCT	Immediate iFR vs. deferred MRI-guided non-culprit PCI (STEMI)	3 years	Death, MI, HF hosp.	No difference: HR 0.95 (0.65–1.40)
DANAMI-3-PRIMULTI	627	RCT	FFR-guided complete vs. IRA-only PCI (STEMI)	10 years	Composite events	FFR-guided complete reduced events: HR 0.76 (*p* = 0.014)
CorMicA	151	RCT	Function testing + stratified Rx vs. standard care (ANOCA)	1 year	Seattle Angina Questionnaire	Improved angina (+22–27%) and QoL
ILIAS ANOCA	153	RCT	Disclosure vs. non-disclosure of function testing (ANOCA)	1 year	SAQ score	SAQSS +9.4 units (*p* = 0.001); ↑ treatment satisfaction
FAITAVI	320	RCT	FFR-guided vs. angio-guided PCI in TAVI	1 year	MACCE	FFR-guided 8.5% vs. 16.0% (HR 0.52, *p* = 0.047)
NOTION-3	455	RCT	PCI + TAVI vs. TAVI alone (FFR ≤ 0.80 or ≥90% DS)	2 years	Death, MI, urgent revasc.	PCI reduced composite (26% vs. 36%, HR 0.71); ↑ bleeding
PRO-TAVI	466	RCT	Deferral of routine PCI vs. PCI in TAVI	1 year	Composite, bleeding	Deferral non-inferior (HR 0.89); lower major bleeding
COMIC-AS	116	Observational (paired pre/post)	FFR/RFR before and after AVR	0.5 year	Reclassification	21.5% lesions cross FFR cutoff after AVR; FFR ≤ 0.83/RFR ≤ 0.85 more accurate pre-AVR

Abbreviations: AVR, aortic valve replacement; CABG, coronary artery bypass grafting; DS, diameter stenosis; FFR, fractional flow reserve; FFRangio, angiography-derived FFR; FU, follow-up; HF, heart failure; HR, hazard ratio; iFR, instantaneous wave-free ratio; IRA, infarct-related artery; MACE, major adverse cardiovascular events; MACCE, major adverse cardiac and cerebrovascular events; MI, myocardial infarction; NI, non-inferiority; OMT, optimal medical therapy; PCI, percutaneous coronary intervention; QFR, quantitative flow ratio; QoL, quality of life; RFR, resting full-cycle ratio; revasc., revascularization; Rx, therapy; SAQ, Seattle Angina Questionnaire; SAQSS, SAQ summary score; STEMI, ST-elevation myocardial infarction; TAVI, transcatheter aortic valve implantation; vFFR, vessel fractional flow reserve; **↑** increase.

## Data Availability

No new data were created or analyzed in this study. Data sharing is not applicable to this article.

## Abbreviations

The following abbreviations are used in this manuscript:

Ach	Acetylcholine
AHA	American Heart Association
AI	Artificial intelligence
ANOCA	Angina with non-obstructive coronary arteries
ATP	Adenosine triphosphate
AUC	Area under the curve
AVR	Aortic valve replacement
CABG	Coronary artery bypass grafting
CAD	Coronary artery disease
CCTA	Coronary computed tomography angiography
CFD	Computational fluid dynamics
CFR	Coronary flow reserve
CFVR	Coronary flow velocity reserve
CI	Confidence interval
CMD	Coronary microvascular dysfunction
COVADIS	Coronary Vasomotion Disorders International Study Group
CT	Computed tomography
CVP	Central venous pressure
DAPT	Dual antiplatelet therapy
DFR	Diastolic hyperemia-free ratio
ECG	Electrocardiogram
ESC	European Society of Cardiology
FFR	Fractional flow reserve
FFRangio	Angiography-derived fractional flow reserve
FFR-CT	Computed tomography-derived fractional flow reserve
FFRMyo	Myocardial fractional flow reserve
HF	Heart failure
HMR	Hyperemic microvascular resistance
HR	Hazard ratio
iFR	Instantaneous wave-free ratio
IMR	Index of microcirculatory resistance
INOCA	Ischemia with non-obstructive coronary arteries
IV	Intravenous
IVUS	Intravascular ultrasound
LAD	Left anterior descending artery
LM	Left main
MACE	Major adverse cardiovascular events
MACCE	Major adverse cardiac and cerebrovascular events
MRI	Magnetic resonance imaging
NHPR	Non-hyperemic pressure ratio
OCT	Optical coherence tomography
OMT	Optimal medical therapy
PCI	Percutaneous coronary intervention
PET	Positron emission tomography
PPG	Pressure pullback gradient
QFR	Quantitative flow ratio
RCA	Right coronary artery
RFR	Resting full-cycle ratio
SAQ	Seattle Angina Questionnaire
SAVR	Surgical aortic valve replacement
SCAI	Society for Cardiovascular Angiography and Interventions
SPECT	Single photon emission computed tomography
STEMI	ST-elevation myocardial infarction
TAVI	Transcatheter aortic valve implantation
vFFR	Vessel fractional flow reserve
VSA	Vasospastic angina
